# A New Approach to Overcome Insulin Resistance in Patients with Impaired Glucose Tolerance: The Results of a Multicenter, Double-Blind, Placebo-Controlled, Randomized Clinical Trial of Efficacy and Safety of Subetta

**DOI:** 10.3390/jcm11051390

**Published:** 2022-03-03

**Authors:** Ashot Mkrtumyan, Alexander Ametov, Tatiana Demidova, Anna Volkova, Ekaterina Dudinskaya, Arkady Vertkin, Sergei Vorobiev

**Affiliations:** 1 Department of Endocrinology, Moscow Clinical Scientific and Practical Center Named after A. S. Loginov, 111123 Moscow, Russia; 2 Department of Endocrinology and Diabetology, A. I. Evdokimov Moscow State University of Medicine and Dentistry, 127473 Moscow, Russia; 3 Department of Endocrinology, Faculty of Medicine, Medical Academy of Continuing Professional Education, 125993 Moscow, Russia; alexander.ametov@gmail.com; 4 Department of Endocrinology, City Clinical Hospital Named after V. P. Demikhova, 117463 Moscow, Russia; t.y.demidova@gmail.com; 5 Department of Endocrinology, Faculty of Medicine, Pirogov Russian National Research Medical University, 117997 Moscow, Russia; 6 Department of Faculty Therapy, Faculty of Medicine, Pavlov First Saint Petersburg State Medical University, 197022 St. Petersburg, Russia; volkovaa@mail.ru; 7 Department of Age-Related Metabolic and Endocrine Disorders, Russian Gerontological Research and Clinical Center, Pirogov Russian National Research Medical University, 129226 Moscow, Russia; katharina.gin@gmail.com; 8 Department of Therapy, Clinical Pharmacology and Emergency Medicine, A. I. Evdokimov Moscow State University of Medicine and Dentistry, 127473 Moscow, Russia; kafedrakf@mail.ru; 9 Department of Therapy, City Clinical Hospital Named after S.I. Spasokukotsky, 127006 Moscow, Russia; 10 Department of Endocrinology, Rostov State Medical University, 344022 Rostov-on-Don, Russia; endocrinrostov@mail.ru

**Keywords:** impaired glucose tolerance, 2-h plasma glucose, glycated hemoglobin

## Abstract

Impaired glucose tolerance (IGT) is a common carbohydrate metabolism disorder world-wide. To evaluate the efficacy and safety of 12-week Subetta therapy in correcting 2-h plasma glucose in patients with IGT, a multicenter, double-blind, placebo-controlled, randomized clinical trial was performed. Derived by technological treatment of antibodies to insulin receptor β-subunit and endothelial NO synthase, Subetta increases the sensitivity of insulin receptors by activating the insulin signaling pathway. Oral glucose tolerance test (OGTT), fasting plasma glucose (FPG), and glycated hemoglobin (HbA1c) were examined at screening, after 4 and 12 weeks. In Per Protocol population, 2-h plasma glucose in the Subetta group decreased by 2.05 ± 2.11 mmol/L (versus 0.56 ± 2.55 mmol/L in the Placebo group) after 12 weeks. The difference between the two groups was 1.49 ± 2.33 mmol/L (*p* < 0.0001). After 12 weeks, 65.2% of patients had 2-h plasma glucose <7.8 mmol/L. FPG remained almost unchanged. HbA1c tended to decrease. The number of adverse events did not differ in both groups. Subetta treatment is beneficial for patients with IGT; it also prevents progression of carbohydrate metabolism disorders.

## 1. Introduction

Impaired glucose tolerance (IGT) is an intermediate stage of carbohydrate metabolism disorders between normal glucose tolerance (NGT) and type 2 diabetes mellitus (T2DM) [1,2]. The average transition time from IGT to T2DM is 4 years [3]. IGT can be associated with impaired fasting glucose (IFG) or normal fasting plasma glucose (FPG) [1,4,5]. IGT is considered as prediabetes, along with IFG and glycated hemoglobin (HbA1c) values in the range of 5.7–6.4% [1,2]. Progression from IGT to T2DM is associated with hyperglycemia, age, and weight [4].

The global prevalence of IGT was 7.5% of the adult population in 2019, and projected prevalence is expected to reach 8.0% in 2030, and 8.6% in 2045 [6].

The rates for the patients who have both IFG and IGT are 15.8% and 20.2% according to World Health Organization (WHO) and American Diabetes Association (ADA) data, respectively [1,5,7].

In patients with IGT, the risk of T2DM development is 6 times higher compared to people with NGT, the relative risk of mortality is 1.48 times more than in a healthy population, and frequency of fatal cardiovascular events is increased by 1.66 times [1]. According to the International Diabetes Federation, 1 in 10 adults are living with diabetes [8].

It was proposed that IGT is a manifestation of insulin resistance due to “lipid overspill from subcutaneous adipose into ectopic sites” [5] (i.e., liver, pancreas, and skeletal muscle) in individuals with a positive energy balance, excess lipid accumulation, and weight gain [9].

Intensive lifestyle/behavior change programs are the first-line interventions in patients with prediabetes for T2DM prevention [10,11,12,13,14,15]. If these approaches are insufficient to achieve weight loss and glycemic control, metformin therapy is recommended by ADA [12]. However, the U.S. Food and Drug Administration has yet to approve any medicines for diabetes prevention [12]. At the same time, in 2021 the European Medicines Agency approved liraglutide [16] and recommended the granting of marketing authorization for semaglutide [17] for weight management in patients with obesity or overweight in the presence of prediabetes and other comorbidities.

One of the approaches to overcome insulin resistance and prevent progression of the carbohydrate metabolism disorders is Subetta therapy. This drug is a biotechnological product containing two components based on affinity-purified antibodies to the β-subunit of the insulin receptor (INSR-β) and to endothelial NO synthase (eNOS). Subetta stimulates activation of the insulin receptor alone and in the presence of insulin increasing phosphorylation of INSR-β [18]. Subetta significantly enhances the insulin sensitivity of human muscle cells through stimulation of glucose transport to myocytes mediated by glucose transporter 4 [19,20].

In patients with T1DM and poor glycemic control in a basal-bolus insulin regimen, Subetta add-on therapy improved the glycemic profile without insulin dose intensification and without increasing the overall hypoglycemia rates [21].

The pharmacological activity of Subetta can be used in patients with IGT to increase the sensitivity of cells to endogenous insulin, which is reduced due to insulin resistance.

In this clinical trial, we evaluated the efficacy and safety of 12-week Subetta therapy in correcting 2-h plasma glucose in patients with IGT.

## 2. Materials and Methods

### 2.1. Study Overview

This multicenter, double-blind, placebo-controlled, randomized, parallel-group clinical trial was carried out between 10 October 2018 and 23 March 2020 in 44 medical institutions in the Russian Federation (see Supplementary Materials, Study overview). The protocol of the study and the study results are posted in the ClinicalTrials.gov results database (NCT03725033) [22].

The screening of patients was performed over 7 days, after which they were randomized into two groups and treated for 12 weeks. Patients visited medical centers three times—on day 1, after 4 weeks (visit 2), and after 12 weeks (visit 3) of the treatment, and they were examined at each visit. The observation period lasted for up to 13 weeks.

### 2.2. Patient Selection and Assessment

Patients of either gender, aged 18–70 years old, with prediabetes, obesity (especially visceral or abdominal obesity), dyslipidemia (with high triglycerides and/or low high-density lipoproteins), arterial hypertension, or high genetic burden of diabetes mellitus (diabetes in first-degree relatives) were considered as candidates for participation in the study.

The screening procedures included medical history, registration of concomitant conditions and diseases, physical examination, calculation of body mass index (BMI), and oral glucose tolerance test (OGTT) for measurement of FPG and 2-h plasma glucose. In addition, venous blood samples (for detection of HbA1c level and biochemical and clinical analyses) and urine samples were obtained from each patient.

All laboratory tests were carried out in the central laboratory, which provided the equipment for collection and sample preparation in the medical centers. Blood samples were collected after a night fast of no less than 10 h. A venous blood sample from the antecubital vein was drawn into two vacuum tubes for plasma and serum separation. The delivery of biological samples was performed within 6 h, in compliance with the requirements of transportation conditions. Measurement of FPG and 2-h plasma glucose was performed using the enzyme (hexokinase) method and photometry in a biochemical analyzer (ARCHITECT c16000, Abbot, Chicago, IL, USA). HbA1c was determined by a method certified in accordance with the National Glycohemoglobin Standardization Program and standardized in compliance with the reference values adopted in the Diabetes Control and Complications Trial. Hematology, blood chemistry and urinalysis were performed to assess the safety of the treatment. Blood chemistry included alkaline phosphatase, aspartate aminotransferase, alanine aminotransferase, creatinine, total cholesterol, total bilirubin, total protein, sodium, and potassium.

The inclusion criteria were as follows: plasma glucose level from 7.8 to 11.0 mmol/L two hours after a 75 g oral glucose load during an OGTT, while FPG < 7.0 mmol/L; HbA1c, 5.7–6.4%; BMI, 25.0–39.9 kg/m^2^; consent to use contraceptive methods during the study (for men and women with reproductive potential).

The recommendations of the WHO and the International Diabetes Federation were used for the diagnosis of IGT and prediabetes.

One of the inclusion criterion was a BMI of 25.0–39.9 kg/m^2^ (overweight, or 1/2 degree obesity). Measurement of body weight and height was carried out at the screening stage on standardized (calibrated according to the factory method) scales and a stadiometer. BMI was calculated according to the formula: weight/(height in meters)^2^.

Candidates with normal BMI were not included in the study. After examination during the screening period, the presence of IGT in patients with a BMI of 25.0–39.9 kg/m^2^ could be not confirmed if the parameters of carbohydrate metabolism did not meet the inclusion criteria.

The exclusion criteria were as follows: T1DM, T2DM, and other specific types of diabetes; acute disease or exacerbation/decompensation of a chronic disease; uncontrolled arterial hypertension; acute coronary syndrome, myocardial infarction, acute impairment of cerebral circulation during the previous 6 months; unstable or life-threatening arrhythmia during the previous 3 months; acute or chronic heart failure with functional class III or IV; respiratory failure; chronic kidney disease (classes C3–5 A3); hepatic insufficiency (class C according to Child–Pugh); oncology disease; an allergy/hypersensitivity to medication administered; mental illness or drug abuse; history of bariatric surgery; pregnancy, breast-feeding; childbirth less than 3 months before enrollment; use of any prohibited medications.

One of the exclusion criterion was uncontrolled arterial hypertension, defined as office systolic blood (BP) pressure ≥ 160 mmHg and/or diastolic BP ≥ 100 mm Hg (grade 2 hypertension or grade 3 hypertension according to ESH/ESC 2013 guidelines for the management of arterial hypertension). BP was measured in the office by a doctor at screening and at every visit using an automated validated upper-arm cuff BP devices based on the oscillometric technique. Measurement of BP was performed in accordance with 2017 American College of Cardiology/American Heart Association Guideline.

### 2.3. Randomization and Blinding

After screening, the patients were randomized into two groups to be assigned Subetta or Placebo. An interactive voice/web response randomization system (based on a random number generator) was used by physicians. Block randomization was performed in blocks of 4. A personal code documented in the chart and unchanged during the study was given to each patient in order to maintain confidentiality.

The studied drug was delivered to medical centers in boxes and packages that did not carry information regarding the active substance. Manufacturing, packaging, and labelling with unique identification codes of the double-blind medications (Subetta or placebo/identical in shape and taste tablet containing excipients) were performed by OOO NPF MMH. Neither participants nor physicians, investigators, trial centers staff, or the Sponsor’s project team were aware of the treatment group assignment throughout the study and until the database lock.

### 2.4. Treatment

Subetta was administered for oral use, 2 tablets per intake twice a day, 15 min before a meal for 12 weeks. The tablet should be held in the mouth until complete dissolution occurred.

Each Subetta tablet contains affinity purified technologically-treated antibodies to β-subunit of the insulin receptor (6 mg) and antibodies to endothelial NO synthase (6 mg). The active substance is produced by patented technology (US Patent 8,617,555 B2) in accordance with European Pharmacopeia requirements [21].

Placebo was administered according to Subetta administration schedule.

The compliance with the study therapies was assessed at the 2nd and 3rd visits according to the count of tablets returned.

Patients were given advice on nutrition and physical activity. A pregnancy test was carried out for all fertile women.

Patients were allowed to use concomitant therapy, including agents acting on the renin-angiotensin system, beta blocking agents, and calcium channel blockers.

Blood glucose lowering drugs, insulins and analogues, corticosteroids, thyroid preparations, hormonal contraceptives, appetite stimulants, anti-obesity preparations, long-action organic nitrates, and diuretics were prohibited drugs.

### 2.5. Study End Points and Statistical Analysis

The primary efficacy end point was a change in 2-h plasma glucose (during OGTT) after 12 weeks of treatment. The secondary end points were as follows: percentage of patients with 2-h plasma glucose <7.8 mmol/L, change in FPG, and change in HbA1c after 12 weeks of treatment.

Statistical analysis was performed using SAS (Version 9.4) (SAS Institute, Cary, NC, USA) statistical software. Data of the full analysis set, excluding the failure to satisfy major entry criteria, were used for the intention-to-treat (ITT) analysis. The data of all patients who completed the therapy as per the study protocol without any missing scheduled visits were used for the Per-Protocol (PP) analysis of the efficacy (data are presented in square brackets).

The sample size was calculated assuming the difference in reduction of 2-h plasma glucose between Product and Placebo groups would be no less than ε = 1.1, while the standard deviation would be σ = 3.11. The power of statistical tests “*p* = (1 − β)” was assumed to be 80% (the probability of correct rejection of the null hypothesis was 0.8); the probability of a type I error “α” was allowed to be less than 0.05% (the probability of the erroneous acceptance of an alternative hypothesis was less than 0.05); the statistical criteria used were two-tailed. The minimum required size for each group was 143 patients; at least 842 patients had to be included, taking into account a dropout rate at 66% subjects (Cw = 0.66) during the study for various reasons. The dropout rate was based on the results of a blinded interim analysis.

Two interim analyses were planned and performed during the study:

(1) Blinded interim analysis to clarify population characteristics and adjust the sample size (only upward). As a result of this analysis, the sample was increased, and Version 2 of the study protocol was released.
(2) Unblinded interim analysis included data from more than 50% of the planned sample (*n* = 538). Unblinded interim analysis was planned for early trial stop due to efficacy (O’Brien–Fleming boundary) or null hypothesis acceptance (Pocock boundary).

According to O’Brien and Fleming [23], if the *p*-value for the primary efficacy endpoint is <0.00388 in the interim analysis of data, the study may be stopped due to evidence of efficacy.

After the inclusion of 538 patients, the study was terminated when interim analysis showed significant reduction in 2-h plasma glucose in patients of the Subetta group in comparison with placebo therapy. Interim analysis demonstrated that the result of the Subetta therapy was sufficient to stop the study due to the achievement of efficacy, because the type I error (0.0028 (<0.0001)) was below the critical value (0.00388) established by the O’Brien–Fleming rules for the interim analysis.

Analysis of continuous variables was carried out using the nonparametric Wilcoxon test and Student’s t-test for normally distributed variables. Normality of variables was accessed using the Shapiro–Wilk test. Multivariate analysis was performed using analysis of variance for repeated measurements (repeated-measures ANOVA, PROC MIXED). The Holm method (PROC MULTTEST) was used as a correction for multiplicity. Fisher’s exact test was used to compare the proportions.

## 3. Results

### 3.1. Patient Demographics and Baseline Characteristics

**Figure 1:**
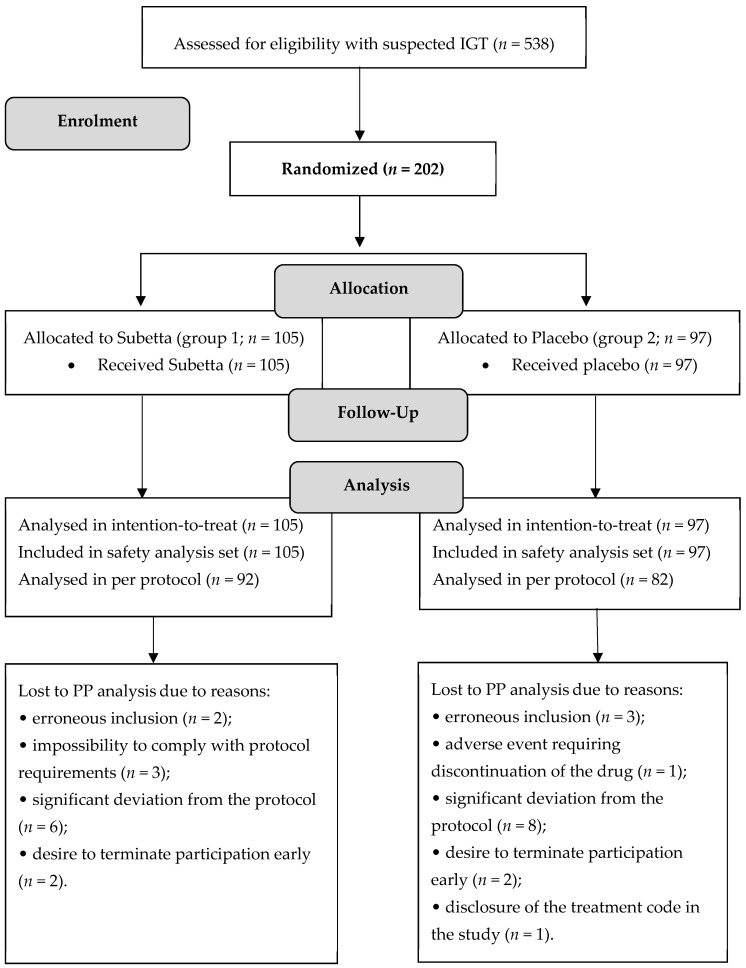
Study design flow diagram.

In total, 538 subjects with suspected IGT were enrolled. After passing the screening procedures, 336 subjects were excluded by the doctors as they did not meet inclusion criteria, or they met exclusion criteria. The remaining 202 subjects were randomized into two groups: 105 to the Subetta group and 97 to the Placebo group. The results of the treatment and observation of these patients (*n* = 202) were used to conduct an ITT analysis of efficacy and to assess the safety of the investigational therapy. The number of patients who received the full course of the therapy, completed all prescribed visits, and did not have significant deviations from the protocol was 174, including 92 in the Subetta group and 82 in the Placebo group. The data from these patients were used for the PP efficacy analysis. Data from 28 patients (13 patients in the Subetta group and 15 patients in the Placebo group) were not included in the PP analysis of efficacy for various reasons. Figure 1 presents the study design flow diagram.

Patients in both groups did not differ in demographic and baseline clinical characteristics, including age, gender, BMI, vital signs, and initial parameters of carbohydrate metabolism (2-h plasma glucose, FPG, and HbA1c). Baseline characteristics of the patients are presented in the Table 1, Table 2 and Table 3.

As can be seen from Table 1, the majority of the study participants were women. To assess the effect of gender on the results of the study, we conducted analysis that showed no statistical significance of gender, both in ITT and PP populations. The statistical analysis used involves mixed models for 2-h plasma glucose with gender covariate and CMH test with Breslow–Day test for percentage of patients with 2-h plasma glucose < 7.8 mmol/L after 12 weeks with gender as the main strata (see Supplementary Materials, Tables S1–S4). 

Various comorbidities were found in 91.4 [91.3]% (*n* = 96 [*n* = 84]) of patients in the Subetta group and in 87.6 [89.0]% (*n* = 85 [*n* = 73]) of subjects of the Placebo group (*p* = 0.49 [*p* = 0.62]).

Sixty one percent of patients [60.9]% (*n* = 62 [*n* = 56]) in the Subetta group and 63.9 [63.4]% (*n* = 62 [*n* = 52]) of patients in the Placebo group (*p* = 1.00 [*p* = 0.76]) had metabolic and nutritional disorders (dyslipidemia, hyperlipidemia, hypertriglyceridemia, hypercholesterolemia, hyperuricemia, etc.); 57.1 [57.6]% (*n* = 60 [*n* = 53]) and 45.4 [47.6]% (*n* = 44 [*n* = 39]) of patients (*p* = 0.12 [*p* = 0.22]) had vascular diseases (arterial hypertension, atherosclerosis of vessels of various localization, chronic venous insufficiency, etc.); 40.0 [42.4]% (*n* = 62 [*n* = 56]) and 37.1 [37.8]% (*n* = 62 [*n* = 56]; *p* = 0.77 [*p* = 0.77 [*p* = 0.64]) had heart diseases (coronary heart disease, angina pectoris, cardiac arrhythmias, NYHA class I/II heart failure, etc.); 22.9 [25.0]% (*n* = 24 [*n* = 23]) and 22.7 [24.4]% (*n* = 22 [*n* = 20]; *p* = 1.00 [*p* = 1.00]) had diseases of muscle, skeletal, and connective tissue; 24.8 [22.8]% (*n* = 26 [*n* = 21]) and 23.7 [25.6]% (*n* = 23 [*n* = 21]; *p* = 0.87 [*p* = 1.00]) had diseases of the gastrointestinal tract; 19.0 [17.4]% (*n* = 20 [*n* = 16]) and 19.6 [18.3]% (*n* = 19 [*n* = 15]; *p* = 1.00 [*p* = 1.00]) had diseases of the liver and biliary tract; 21.0 [21.7]% (*n* = 22 [*n* = 20]) and 20.6 [20.7]% (*n* = 20 [*n* = 17]; *p* = 1.00 [*p* = 1.00]) had thyroid gland pathology. Other diseases were less common. Statistical analysis using Fisher’s exact test did not reveal significant differences between groups of patients in the incidence of concomitant diseases.

Seventy two percent [71.7]% (*n* = 75 [*n* = 66]) of patients in the Subetta group and 72.2 [72.0]% (*n* = 70 [*n* = 59]) in the Placebo group received permitted concomitant therapy (*p* = 1.00 [*p* = 1.00]). Most patients in both groups took drugs for the treatment of cardiovascular diseases, including agents acting on the renin-angiotensin system (52.4 [53.3]% (*n* = 55 [*n* = 49]) in the Subetta group and 53.6 [52.4]% (*n* = 52 [*n* = 43]) in the Placebo group; *p* = 0.89 [*p*] = 1.00]), beta blocking agents (30.5 [29.3]% (*n* = 32 [*n* = 27]) and 26.8 [29.3]% (*n* = 26 [*n* = 24]); *p* = 0.64 [*p* = 1.00]), calcium channel blockers (13.3 [15.2]% (*n* = 14 [*n* = 14]) and 10.3 [9.8]% (*n* = 10 [*n* = 8]), respectively; *p* = 0.52 [*p* = 0.36]). Medications from other pharmacological groups were taken by a relatively small percentage of the study participants. Fisher’s exact test did not show differences between groups for concomitant therapy.

Patients in the Subetta and the Placebo groups excluded from the PP analysis also did not differ (both among themselves and compared to patients whose data were included in the analysis) in baseline demographic, anthropometric, clinical characteristics, comorbidities, and concomitant therapy.

The adherence of patients to the therapy after 4 weeks was 100.5 ± 12.5 [99.7 ± 5.6]% in the Subetta group and 101.9 ± 13.6 [100.6 ± 4.8]% in the Placebo group, and after 12 weeks of treatment this was 98.1 ± 7.4% [98.3 ± 4.8]% and 100.5 ± 8.5 [98.8 ± 4.4]%, respectively (*p* = 0.76 [*p* = 0.72]).

### 3.2. Primary Efficacy Endpoint

**Figure 2:**
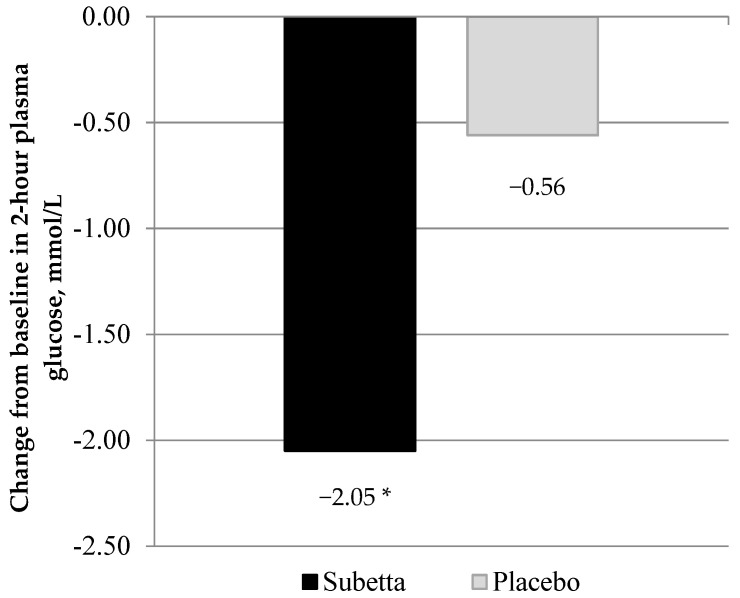
Change from baseline in 2-h plasma glucose after 12 weeks of treatment (PP analysis). Note. * *p* < 0.0001 vs. placebo.

Two-hour plasma glucose concentration in the Subetta group significantly decreased after 12 weeks of treatment. Figure 2 indicates the change from baseline of 2-h plasma glucose after 12 weeks of treatment in patients of the PP sample.

The difference in 2-h glucose level between baseline and after 12 weeks of treatment in the Subetta group was −1.9 ± 2.2 [−2.0 ± 2.1] mmol/L (versus −0.7 ± 2.2 [−0.5 ± 2.5] mmol/L in the Placebo group). The difference in the reduction of 2-h plasma glucose between the Subetta and the Placebo groups was 1.2 ± 2.3 [1.4 ± 2.3] mmol/L (*p* = 0.0028 [*p* < 0.0001]).

### 3.3. Secondary Efficacy Endpoint

**Figure 3:**
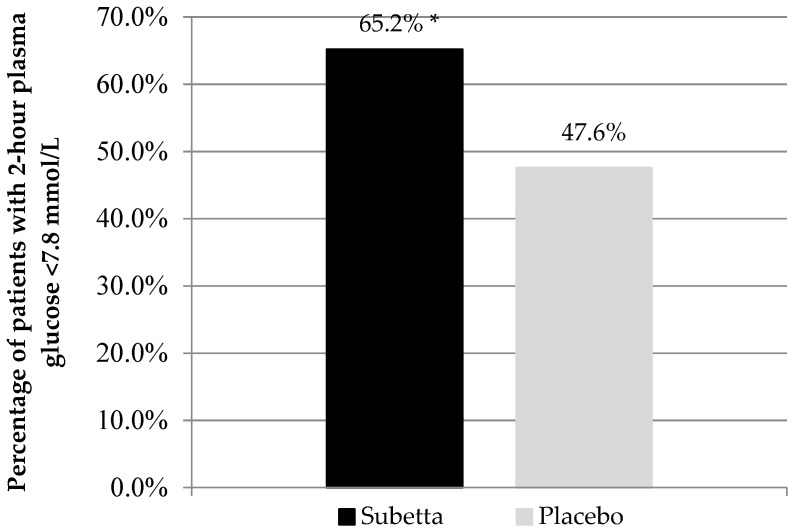
Percentage of patients with 2-h plasma glucose < 7.8 mmol/L after 12 weeks of treatment (PP analysis). Note. * *p* = 0.0219 vs. placebo.

Figure 3 shows the percentage of patients with 2-h plasma glucose < 7.8 mmol/L after 12 weeks of treatment in patients of the PP sample, where the advantage of Subetta was 17.6% ([*p* = 0.0219]).

The baseline FPG level was either within the normal range or slightly exceeded the normal threshold level of 6.0 mmol/L in most patients in both groups. The boundaries of the lower and upper quartiles were 5.4–6.3 [5.4–6.3] mmol/L in the Subetta group and 5.5–6.3 [5.5–6.3] mmol/L in the Placebo group (*p* = 0.06 [*p* = 0.08]).

FPG remained almost unchanged during 12 weeks of treatment in both groups. The boundaries of the lower and upper quartiles after 12 weeks were 5.4–6.5 [5.4–6.5] mmol/L and 5.4–6.4 [5.4–6.5] mmol/L in the Subetta and the Placebo group, respectively (*p* = 0.99 [*p* = 0.74]).

Within 12 weeks of treatment, there was a trend towards a decrease in HbA1c level in both groups. The boundaries of the lower and upper quartiles after 12 weeks were 5.5–6.0 [5.5–6.0]% and 5.6–6.0 [5.6–6.1]% in the Subetta and the Placebo group, respectively. This means that, after 12 weeks, 25% of patients in the Subetta group had HbA1c below 5.5%.

### 3.4. Safety Analysis

Subetta had no impact on the vital signs of patients, including blood pressure, heart rate, and respiratory rate. The mean values of the vital signs throughout the study were normal and well-controlled. There were no differences in these parameters during treatment between both groups.

In total, 16 adverse events (AEs) were reported in 15 (14.3%) patients of the Subetta group, and 27 AEs reported in 20 (20.6%) patients of the Placebo group (see Supplementary Table S5).

Frequency analysis (Fisher’s exact test) did not reveal significant differences between the number of patients with AEs in the Subetta and the Placebo groups (*p* = 0.27).

The most frequent AEs were changes in laboratory and instrumental test results. In the Subetta group, this was an increase in the number of leukocytes in urine (*n* = 1), an increase in alanine aminotransferase (ALT) level (*n* = 1), an increase in blood pressure (*n* = 1), weight loss (*n* = 1), and in the Placebo group, an increase in the ALT (*n* = 1), an increase in aspartate aminotransferase (*n* = 1), an increase in HbA1c level (*n* = 1), an increase in blood pressure (*n* = 3), and a decrease in weight (*n* = 1).

Within 3 months, in 7 patients IGT progressed to T2DM, including 2 patients in the Subetta group and 5 patients in the Placebo group.

In the Subetta group, 13 (81.43%) AEs were mild and 3 (18.7%) were moderate. No AEs were reported with definite/possible relationship with the study drug. The frequency of distribution of AEs depending on the severity (*p* = 0.42) and the relationship with the drug (*p* = 0.17) did not differ in the two groups (see Supplementary Tables S6 and S7).

AEs classified as severe were not registered in the clinical trial. There was no evidence of drug-to-drug interaction with medications administered concomitantly with Subetta, nor were there any hypersensitivity events.

## 4. Discussion and Conclusions

This study demonstrated the efficacy of Subetta in patients with IGT. The therapeutic action of Subetta is manifested in the reduction of 2-h glucose, which prevents progression of disorders of carbohydrate metabolism. After 12 weeks of Subetta administration, 2-h glucose was restored to normal levels in most patients with IGT.

Administration of Subetta had no effect on FPG. On the one hand, this may indicate that the drug has (first of all) an antihyperglycemic effect, without affecting normal plasma glucose concentration. On the other hand, it was found that disorders of carbohydrate metabolism in IFG and IGT differ in development mechanisms. It is known that impaired insulin secretion and suppression of gluconeogenesis are the main mechanisms in the development of IFG [24]. Obviously, the pharmacological activity of Subetta, which consists of sensitizing the insulin receptor and increasing the sensitivity of cells to insulin, does not significantly affect the processes of hormone secretion and gluconeogenesis [20]. In this regard, the level of fasting glycemia in patients who took the study drug for 12 weeks remained unchanged, while 2-h (post-load) hyperglycemia was amenable to good correction (precisely by reducing insulin resistance).

Along with eliminating IGT in most patients, Subetta “initiated” HbA1c correction. Baseline “pre-diabetic” HbA1c values tended to decrease after 12 weeks of treatment. As is known, HbA1c is a long-term blood glucose control indicator; it reflects glucose concentration over the past 2–3 months [25]. Obviously, during the very first weeks after starting the treatment, there was no significant change in 2-h plasma glucose. Therefore, peaks of postprandial hyperglycemia in this initial treatment period contributed negatively and HbA1c was not decreased below 5.7% in most patients. However, it should be noted that 25% of patients in the Subetta group had HbA1c values below 5.5%. It is possible that the use of the drug for a longer period may normalize HbA1c in most patients with IGT.

Insulin resistance, which is leading in carbohydrate metabolism disorders in patients with IGT, is well corrected by Subetta over a 12-week course of treatment. The effect of the drug is realized by improving glucose utilization due to a decrease in insulin resistance. Obviously, longer therapy can contribute to a more significant normalization of carbohydrate metabolism and prevent the progression of disorders to T2DM.

The endothelioprotective effect of the second component of Subetta (technologically treated antibodies to eNOS) [19] is also important in therapy because vascular complications manifest already at the IGT stage [26]. The synergistic action of the two components of the drug has a positive effect on both insulin resistance and endothelial dysfunction, thereby preventing the progression of micro- and macroangiopathies.

This study has several limitations. It has a relatively small sample size due to a high drop-out rate during screening. Patients with serious concomitant diseases were not included in the study, and therefore the efficacy of Subetta in these patient categories was not investigated. Due to the short duration of treatment (12 weeks), the effects of Subetta on body weight, as well as waist circumference and lipid profile, were not evaluated. In addition, the therapeutic effects of various schemes of Subetta administration have not been evaluated.

In conclusion, the results of this study demonstrated the therapeutic potential of Subetta in patients with IGT. Obviously, longer studies need to be planned to prove the long-term effects of Subetta in patients with insulin resistance.

## 5. Patents

Subetta is a drug manufactured by OOO “NPF” MATERIA MEDICA HOLDING”. Patents on Subetta: US 8,617,555 B2; MX 331246; RU 2509572, and RU2531048.

## Figures and Tables

**Table 1:** Baseline demographic and anthropometric characteristics of the patients.

Characteristics	ITT Analysis (N = 202)		PP Analysis (N = 174)	
	Subetta	Placebo	Subetta	Placebo
Age, years				
Mean ± SD	56.6 ± 8.6	56.1 ± 8.6	57.0 ± 9.1	56.3 ± 8.6
Median	58	57	58.5	56.5
Minimum	28	33	28	38
Maximum	70	69	70	69
Q1–Q3	51–54	52–64	52.5–64	52–64
Statistics	Z = 0.64; *p* = 0.52		Z = 0.84; *p* = 0.40	
Male/female, %	25.7/74.3	22.7/77.3	27.2/72.8	25.6/74.4
	*p* = 0.63		*p* = 0.86	
Body weight, kg				
Mean ± SD	88.2±15.0	88.7±14.5	87.8±14.4	89.3 ± 14.8
Median	85	89	84.8	89
Minimum	58	62	58	62
Maximum	120	128.2	120	128.2
Q1–Q3	76.6–99.1	77–98	77.3 – 98.4	77–98.7
Statistics	Z = 0.30; *p* = 0.76		Z = 0.68; *p* = 0.50	
BMI, kg/m^2^				
Mean ± SD	31.8 ± 4.2	32.0 ± 4.3	31.7 ± 4.0	32.0 ± 4.1
Median	31.2	32.1	31.2	31.9
Minimum	25.1	25.3	25.1	25.4
Maximum	39.7	39.5	39.7	39.5
Q1–Q3	28.4–35	28.2–35.5	28.6–34.7	28.3–35.5
Statistics	Z = 0.28; *p* = 0.78		Z = 0.30; *p* = 0.77	

Notes. Mean ± SD—mean and standard deviation. Q1–Q3—the first and third quartiles. N—number of patients. The age of the patients was analyzed using the Wilcoxon test; the result of the normality test using the Shapiro–Wilk test: ITT—Subetta—*p* = 0.0003, Placebo—*p* = 0.0019; PP—Subetta—*p* = 0.0002, Placebo—*p* = 0.0030. Gender was analyzed and compared using Fisher’s exact test. The result of the normality test using the Shapiro–Wilk test: BMI—Subetta—*p* = 0.0006, Placebo—*p* = 0.0017; PP—Weight—Subetta—*p* = 0.1249, Placebo—*p* = 0.2565, BMI—Subetta—*p* = 0.0055, Placebo—*p* = 0.0008.

**Table 2:** Baseline blood pressure of the patients.

	ITT Analysis (N = 202)		PP Analysis (N = 174)	
	Subetta	Placebo	Subetta	Placebo
Systolic blood pressure, mm Hg				
Mean ± SD	127.5 ± 8.0	127.8 ± 8.5	127.4 ± 7.9	127.8 ± 8.3
Median	129	127	128.5	127
Minimum	100	98	100	98
Maximum	147	156	147	156
Q1–Q3	122–132	122–134	122–132	122–134
Statistics	Z = 0.48; *p* = 0.63		Z = 0.56; *p* = 0.58	
Diastolic blood pressure, mm Hg				
Mean ± SD	79.1 ± 5.6	79.4 ± 6.9	78.8 ± 5.7	79.4 ± 7.0
Median	80	80	80	80
Minimum	62	54	62	54
Maximum	90	97	90	97
Q1–Q3	75–83	75–84	74.5–83	75–84
Statistics	Z = 0.15; *p* = 0.88	Z = 0.41; *p* = 0.68	Z = 0.15; *p* = 0.88	Z = 0.41; *p* = 0.68

Notes. Mean ± SD—mean and standard deviation. Q1–Q3—the first and third quartiles. N—number of patients. Blood pressure indicators were analyzed using Student’s t-test and Wilcoxon’s test; the result of the normality test using the Shapiro–Wilk test: Systolic blood pressure (ITT analysis)—Subetta—*p* = 0.1900, Placebo—*p* = 0.0071; (PP analysis)—Subetta—*p* = 0.1205, Placebo—*p* = 0.0019; Diastolic blood pressure (ITT analysis)—Subetta—*p* = 0.0483, Placebo—*p* = 0.2262; (PP analysis)—Subetta—*p* = 0.0489, Placebo—*p* = 0.2045.

**Table 3:** Baseline parameters of carbohydrate metabolism of the patients.

Parameters	ITT Analysis (N = 193 *)		PP Analysis (N = 174)	
	Subetta	Placebo	Subetta	Placebo
2-h plasma glucose, mmol/L				
Mean ± SD	9.3 ± 0.9	9.1 ± 0.9	9.3 ± 0.8	9.1 ± 0.9
Median	9.2	9.0	9.2	9.0
Q1–Q3	8.5–9.9	8.4–9.6	8.5–9.9	8.4–9.6
95% CI	9.1–9.4	8.9–9.3	9.1–9.4	8.9–9.3
N *	101	92	92	82
Statistics	Z = 1.22; *p* = 0.22		Z = 1.47; *p* = 0.14	
Fasting plasma glucose, mmol/L				
Mean ± SD	5.8 ± 0.6	5.9 ± 0.6	5.9 ± 0.6	5.9 ± 0.6
Median	5.9	5.9	5.95	5.9
Q1–Q3	5.4–6.3	5.5–6.3	5.4–6.3	5.5–6.3
95% CI	5.7–6.0	5.7–6.0	5.7–6.0	5.7–5.9
N *	101	92	92	82
Statistics	Z = 0.29; *p* = 0.77		Z = 0.23; *p* = 0.82	
HbA1c,%				
Mean ± SD	6.0 ± 0.2	6.0 ± 0.20	6.0 ± 0.2	6.0 ± 0.2
Median	5.9	6.0	5.9	6.0
Q1–Q3	5.8–6.1	5.8–6.2	5.8–6.1	5.8–6.2
95% CI	5.9–6.0	6.0–6.0	5.9–6.00	6.0–6.0
N *	101	92	92	82
Statistics	Z = 1.92; *p* = 0.06		Z = 1.72; *p* = 0.08	

Notes. Mean ± SD—mean and standard deviation. Q1–Q3—the first and third quartiles. * N—number of patients. Blood samples were taken from 9 randomized patients (n = 4, Subetta group; n = 5, placebo group) who were fully treated and underwent all procedures according to the protocol, but the central laboratory did not provide carbohydrate metabolism values due to technical problems. Data were analyzed using the Wilcoxon test; the result of the normality test using the Shapiro–Wilk test: 2-h plasma glucose (ITT analysis)—Subetta—*p* = 0.0013, placebo—*p* = 0.0034; (PP analysis)—Subetta—*p* = 0.0047, Placebo—*p* = 0.0050; fasting plasma glucose (ITT analysis)—Subetta—*p* = 0.0002, Placebo—*p* = 0.0226; (PP Analysis)—Subetta—*p* = 0.0001, Placebo—*p* = 0.0490; HbA1c (ITT analysis)—Subetta—*p* = 0.0001, Placebo—*p* = 0.0009; (PP Analysis)—Subetta—*p* = 0.0001, Placebo—*p* = 0.0011.

## Data Availability

Study details are provided at (https://clinicaltrials.gov/ct2/show/NCT03725033 (accessed on 23 January 2022)) and can also be obtained by contacting the study sponsor OOO “NPF” MATERIA MEDICA HOLDING”.

## Supplementary Materials

The following are available online at https://www.mdpi.com/article/10.3390/jcm11051390/s1: Study overview; Table S1: Level of 2-h plasma glucose with gender covariate (ITT analysis); Table S2: Level of 2-h plasma glucose with gender covariate (PP analysis); Table S3: Percentage of patients with 2-h plasma glucose <7.8 mmol/L after 12 weeks of treatment (ITT analysis); Table S4: Percentage of patients with 2-h plasma glucose <7.8 mmol/L after 12 weeks of treatment (PP analysis); Table S5: Adverse events; Table S6: Relationship between the drug and adverse events; Table S7: Adverse events severity.
