# Supplementary material for: A New Approach to Overcome Insulin Resistance in Patients with Impaired Glucose Tolerance: The Results of a Multicenter, Double-Blind, Placebo-Controlled, Randomized Clinical Trial of Efficacy and Safety of Subetta
Source: J Clin Med. 2022 Mar 3;11(5):1390. doi: 10.3390/jcm11051390 (PMC8910875; doi:10.3390/jcm11051390)
Supplement: Supplementary file 1 [file jcm-11-01390-s001.zip › jcm-1589103-supplementary.pdf]

**Supplementary Materials**

**Study overview**

The multicenter, double-blind, placebo-controlled, randomized, parallel-group clinical trial was carried out between October 10, 2018 and March 23, 2020 in 44 medical institutions in the Russian Federation. The following centers were selected from across the Russian Federation: Arkhangelsk Regional Clinical Hospital, Arkhangelsk; Belgorod State National Research University, Belgorod; Ural State Medical University, Ekaterinburg; City Clinical Hospital 40, Ekaterin-burg; Central City Hospital 7, Ekaterinburg; Kirov Clinical Hospital 7 named. V.I. Yurlova, Kirov; Kuban State Medical University, Krasnodar; Regional Clinical Hospital 1 named after Professor Ochapovsky, Krasnodar; Clinic of Professor Gorbakov. Krasnogorsk; Krasnogorsk city hospital 1, Krasnogorsk; City Clinical Hospital named after F.I. Inozemtseva Department of Health of Moscow, Moscow; City Clinical Hospital named after V.P. Demikhova, Moscow; Moscow Clinical Scientific and Practical Center of the Moscow City Health Department, Moscow; The Central Clinical Hospital of the Russian Academy of Sciences, Moscow; Pirogov Russian National Research Medical University, Moscow; Polyclinic 5 of the Administrative Department of the President of the Russian Federation, Moscow; National Medical Research Center for Rehabilitation and Balneology, Moscow; Clinic "Bessalar", Moscow; Moscow State University of Medicine and Dentistry, Moscow; City Clinical Hospital 10 of the Kanavinsky District of Nizhny Novgorod, Nizhny Novgorod; Semashko Nizhny Novgorod Regional Clinical Hospital, Nizhny Novgorod; Medical Center "Healthy Family", Novosibirsk; Institute of Cytology and Genetics of the Russian Academy of Sciences,Novosibirsk; Novosibirsk State Medical University, Novosibirsk; Rostov State Medical University, Rostov-on-Don; Polyclinic Complex JSC, Saint Petersburg; City Clinical Hospital 109, Saint Petersburg; City Polyclinic 25, Saint Petersburg; Military Medical Academy named after S.M. Kirov Defense Ministry of the RF, Saint Petersburg; City Polyclinic No. 117, Saint Petersburg; City Polyclinic № 54, Saint Petersburg; Road Clinical Hospital JSC Russian Railways, Saint Petersburg; Pavlov First Saint Petersburg State Medical University, Saint Petersburg; Limited Liability Company Gastroenterologichesky Center Expert, Saint Petersburg; City Polyclinic № 106, Saint Petersburg; Pokrovskaya City Hospital, Saint Petersburg; City Polyclinic №4, Saint Petersburg; Samara city hospital 4, Samara; Center "Diabetes", Samara; Saratov State Medical University named after V. I. Razumovsky, Saratov; Volgograd State Medical University, Volgograd; Voronezh Regional Clinical Consultative and Diagnostic Center; Medical Consultations and Research Center “Practice”, Yaroslavl; Regional Clinical Hospital, Yaroslavl.

Table S1. Level of 2-hour plasma glucose with gender covariate (ITT analysis).

| | **Male** | | **Female** | |
|---|---|---|---|---|
| | **Subetta** | **Placebo** | **Subetta** | **Placebo** |
| **Baseline** | | | | |
| M ± SD | 9.27±0.89 | 8.85±0.87 | 9.27±0.88 | 9.19±0.85 |
| Median | 9.1 | 8.8 | 9.2 | 9.1 |
| Q1 – Q3 | 8.5 – 10.2 | 8.1 – 9.4 | 8.5 – 9.8 | 8.4 – 9.7 |
| 95% CI | 8.91 – 9.62 | 8.45 – 9.25 | 9.07 – 9.47 | 8.99 – 9.39 |
| N | 27 | 21 | 74 | 71 |
| **12 weeks** | | | | |
| M ± SD | 7.37±2.58 | 7.85±2.28 | 7.25±1.87 | 8.46±2.48 |
| Median | 6.4 | 8.0 | 7.15 | 7.8 |
| Q1 – Q3 | 5.6 – 10.1 | 5.7 – 9.5 | 5.8 – 8.6 | 6.6 – 9.9 |
| 95% CI | 6.35 – 8.40 | 6.81 – 8.88 | 6.82 – 7.68 | 7.88 – 9.05 |
| N | 27 | 21 | 74 | 71 |
| **Δ between baseline and 12 weeks** | | | | |
| M ± SD | -1.89±2.56 | -1.00±2.45 | -2.02±2.09 | -0.73±2.59 |
| Median | -2.9 | -0.5 | -1.9 | -1.4 |
| Q1 – Q3 | -3.9 – 0.40 | -3.1 – 0.60 | -3.6 – -0.70 | -2.4 – 0.7 |
| 95% CI | -2.91 – -0.88 | -2.12 – 0.12 | -2.50 – -1.54 | -1.34 – -0.11 |
| N | 27 | 21 | 74 | 71 |
| **Δ between Subetta and Placebo** | | | | |
| M ± SD | 0.89 ± 2.52 | | 1.29 ± 2.35 | |
| 95% CI | -0.58 – 2.37 | | 0.52 – 2.06 | |
| Statistics | Gender $F_{1/190}$=0.02; p=0.8944<br>Group $F_{1/190}$=12.06; p=0.0006 | | | |

Table S2. Level of 2-hour plasma glucose with gender covariate (PP analysis).

| | Male | | Female | |
|---|---|---|---|---|
| | **Subetta** | **Placebo** | **Subetta** | **Placebo** |
| **Baseline** | | | | |
| M ± SD | 9.31±0.90 | 8.85±0.87 | 9.26±0.85 | 9.16±0.84 |
| Median | 9.2 | 8.8 | 9.2 | 9.1 |
| Q1 – Q3 | 8.5 – 10.2 | 8.1 – 9.4 | 8.5 – 9.8 | 8.5 – 9.6 |
| 95% CI | 8.94 – 9.69 | 8.45 – 9.25 | 9.06 – 9.47 | 8.95 – 9.38 |
| N | 25 | 21 | 67 | 61 |
| **12 weeks** | | | | |
| M ± SD | 7.21±2.61 | 7.85±2.28 | 7.23±1.80 | 8.75±2.51 |
| Median | 6.3 | 8.0 | 7.2 | 8.2 |
| Q1 – Q3 | 5.6 – 10.0 | 5.7 – 9.5 | 5.8 – 8.6 | 6.9 – 10.2 |
| 95% CI | 6.13 – 8.29 | 6.81 – 8.88 | 6.79 – 7.67 | 8.11 – 9.40 |
| N | 25 | 21 | 67 | 61 |
| **Δ between baseline and 12 weeks** | | | | |
| M ± SD | -2.10±2.55 | -1.00±2.45 | -2.03±1.95 | -0.41±2.59 |
| Median | -3.2 | -0.5 | -1.7 | -1.0 |
| Q1 – Q3 | -3.9 – 0.20 | -3.1 – 0.60 | -3.3 – -0.70 | -2.1 – 0.9 |
| 95% CI | -3.15 – -1.05 | -2.12 – 0.12 | -2.51 – -1.56 | -1.07 – 0.26 |
| N | 25 | 21 | 67 | 61 |
| **Δ between Subetta and Placebo** | | | | |
| M ± SD | 1.10 ± 2.51 | | 1.63 ± 2.27 | |
| 95% CI | -0.39 – 2.59 | | 0.83 – 2.42 | |
| Statistics | Gender $F_{1/171}$=0.60; p=0.4403<br>Group $F_{1/171}$=17.63; p<0.0001 | | | |

Table S3. Percentage of patients with 2-hour plasma glucose <7.8 mmol/L after 12 weeks of treatment (ITT analysis).

| | Male | | Female | |
|---|---|---|---|---|
| | **Subetta (N=27)** | **Placebo (N=21)** | **Subetta (N=74)** | **Placebo (N=71)** |
| Percentage of patients | 17 (63.0) | 10 (47.6) | 47 (63.5) | 36 (50.7) |
| Statistics | Breslow-day: p=0.8836<br>CMH Value =3.5; p=0.0614 | | | |

Table S4. Percentage of patients with 2-hour plasma glucose <7.8 mmol/L after 12 weeks of treatment (PP analysis).

| | Male | | Female | |
|---|---|---|---|---|
| | **Subetta (N=25)** | **Placebo (N=21)** | **Subetta (N=67)** | **Placebo (N=61)** |
| Percentage of patients | 17 (68.0) | 10 (47.6) | 43 (64.2) | 29 (47.5) |
| Statistics | Breslow-day: p=0.8137<br>CMH Value =5.43; p=0.0198 | | | |

Table S5: Adverse events

| **SOC / Adverse event** | **Subetta (N=105)** | **Placebo (N=97)** | **p-value** |
|---|---|---|---|
| **Number of subjects reporting at least one AE *** | **15 (14.3)** | **20 (20.6)** | **0.27** |
| **Total AEs** | **16** | **27** | |

| SOC / Adverse event | Subetta (N=105) | Placebo (N=97) | p-value |
|---|---|---|---|
| **Gastrointestinal disorders** | | | |
| At least one event | **1 (1.0)** | **2 (2.1)** | **0 .61** |
| Abdominal pain | 0 (0.0) | 2 (2.1) | |
| Vomiting | 1 (1.0) | 1 (1.0) | |
| **Infections and infestations** | | | |
| At least one event | **3 (2.9)** | **7 (7.2)** | **0.20** |
| Urinary tract infection | 0 (0.0) | 1 (1.0) | |
| Acute respiratory tract infection | 1 (1.0) | 3 (3.1) | |
| Bronchitis | 0 (0.0) | 1 (1.0) | |
| Conjunctivitis | 1 (1.0) | 0 (0.0) | |
| Cystitis | 0 (0.0) | 1 (1.0) | |
| Rhinitis | 1 (1.0) | 0 (0.0) | |
| Nasopharyngitis | 0 (0.0) | 1 (1.0) | |
| **Changes in laboratory and instrumental parameters** | | | |
| At least one event | **3 (2.9)** | **5 (5.2)** | **0.48** |
| Increased white blood cells in the urine | 1 (1.0) | 0 (0.0) | |
| Increased alanine aminotransferase | 1 (1.0) | 1 (1.0) | |
| Increased aspartate aminotransferase | 0 (0.0) | 1 (1.0) | |
| Increase in glycated hemoglobin | 0 (0.0) | 1 (1.0) | |
| Increased blood pressure | 1 (1.0) | 3 (3.1) | |
| Weight loss | 1 (1.0) | 1 (1.0) | |
| **Metabolic and nutrition disorders** | | | |
| At least one event | **2 (1.9)** | **6 (6.2)** | **0.16** |
| Postprandial hyperglycemia | 0 (0.0) | 1 (1.0) | |
| Diabetes mellitus | 2 (1.9) | 5 (5.2) | |
| **Respiratory, Chest and Mediastinal Disorders** | | | |
| At least one event | **1 (1.0)** | **0 (0.0)** | **1.00** |
| Nose bleeding | 1 (1.0) | 0 (0.0) | |
| **Muscle, skeletal and connective tissue disorders** | | | |
| At least one event | **2 (1.9)** | **0 (0.0)** | **0.50** |
| Knee pain | 1 (1.0) | 0 (0.0) | |
| Backache | 1 (1.0) | 0 (0.0) | |
| **Nervous system disorders** | | | |
| At least one event | **1 (1.0)** | **2 (2.1)** | **0.61** |
| Headache | 1 (1.0) | 2 (2.1) | |
| **General disorders** | | | |
| At least one event | **2 (1.9)** | **0 (0.0)** | **0.50** |
| Deterioration | 1 (1.0) | 0 (0.0) | |
| Unsteadiness of gait | 1 (1.0) | 0 (0.0) | |
| **Injury, poisoning and complications of procedures** | | | |
| At least one event | **0 (0.0)** | **2 (2.1)** | **0.23** |
| Brain concussion | 0 (0.0) | 1 (1.0) | |
| Bruised thigh | 0 (0.0) | 1 (1.0) | |

*Note*. AE – adverse event. SOC – system-organ-class in accordance with Medical Dictionary for Regulatory Activities (MedDRA). * Differences in the number of patients with at least one AE and the total number of AEs in the group are due to the fact that in some patients there were 2, or 3 AEs registered.

Table S6: Relationship between the drug and adverse events

| Relationship | Subetta (N=105), n (%) | Placebo (N=97), n (%) | p-value * |
|---|---|---|---|
| Unrelated | 15 (93.7) | 24 (88.9) | 0.166 |
| Uncertain | 0 (0.0) | 1 (3.7) | |
| Possible | 1 (6.3) | 2 (7.4) | |
| **Total** | **16(100.0)** | **27 (100.0)** | |

*Note.* * The results of the Fisher's exact test.

Table S7: Adverse events severity

| Severity | Subetta (N=105), n (%) | Placebo (N=97), n (%) | p-value * |
|---|---|---|---|
| Mild | 13 (81.3) | 24 (88.9) | 0.419 |
| Moderate | 3 (18.7) | 3 (11.1) | |
| **Total** | **16 (100.0%)** | **27 (100.0%)** | |

*Note.* * The results of the Fisher's exact test.
